# Dual-energy spectral CT characteristics in surgically resected lung adenocarcinoma: comparison between Kirsten rat sarcoma viral oncogene mutations and epidermal growth factor receptor mutations

**DOI:** 10.1186/s40644-019-0261-1

**Published:** 2019-11-29

**Authors:** Meng Li, Li Zhang, Wei Tang, Jian-Chun Duan, Yu-Jing Jin, Lin-Lin Qi, Ning Wu

**Affiliations:** 1 Department of Diagnostic Radiology, National Cancer Center/National Clinical Research Center for Cancer/Cancer Hospital, Chinese Academy of Medical Sciences and Peking Union Medical College, Beijing, 100021, China; 2 Department of Medical Oncology, National Cancer Center/National Clinical Research Center for Cancer/Cancer Hospital, Chinese Academy of Medical Sciences and Peking Union Medical College, Beijing, 100021, China; 3 PET-CT Center, National Cancer Center/National Clinical Research Center for Cancer/Cancer Hospital, Chinese Academy of Medical Sciences and Peking Union Medical College, Beijing, 100021, China

**Keywords:** Dual-energy spectral computed tomography, Adenocarcinoma of lung, Solid nodule, Subsolid nodule, *EGFR* mutation, *KRAS* mutation

## Abstract

**Background:**

Kirsten rat sarcoma viral oncogene homolog (*KRAS*) and epidermal growth factor receptor (*EGFR*) are the two most frequent and well-known oncogene of lung adenocarcinoma. The purpose of this study is to compare the characteristics measured with dual-energy spectral computed tomography (DESCT) in lung adenocarcinoma patients who have *KRAS* and *EGFR* gene mutations.

**Methods:**

Patients with surgically resected lung adenocarcinoma (*n* = 72) were enrolled, including 12 patients with *KRAS* mutations and 60 patients with *EGFR* mutations. DESCT quantitative parameters, including the CT number at 70 keV, the slopes of the spectral attenuation curves (slope λ HU), normalized iodine concentration (NIC), normalized water concentration (NWC), and effective atomic number (effective Z), were analyzed. A multiple logistic regression model was applied to discriminate clinical and DESCT characteristics between the types of mutations.

**Results:**

The *KRAS* mutation was more common in people who smoked than the *EGFR* mutation. Nodule type differed significantly between the *KRAS* and *EGFR* groups (*P* = 0.035), and all *KRAS* mutation adenocarcinomas were solid nodules. Most DESCT quantitative parameters differed significantly between solid nodules and subsolid nodules. CT number at 70 keV, slope λ HU, NIC, and effective Z differed significantly between the *KRAS* and *EGFR* groups (*P* = 0.006, 0.017*,* 0.013 and 0.010) with solid lung adenocarcinoma. Multivariate logistic analysis of DESCT and clinical features indicated that besides smoking history, the CT value at 70 keV (OR = 0.938, *P* = 0.009) was significant independent factor that could be used to differentiate *KRAS* and *EGFR* mutations in solid lung adenocarcinoma.

**Conclusions:**

DESCT would be a potential tool to differentiate lung adenocarcinoma patients with a *KRAS* mutation from those with an *EGFR* mutation.

## Introduction

Lung cancer is the leading cause of cancer deaths worldwide, and adenocarcinoma is its most common histologic form [1, 2]. Lung adenocarcinoma is considered a highly molecular heterogeneous disease [3]. In recent years, interest in the key role of proto-oncogenes in lung adenocarcinoma has been growing because of the rapid advances in molecularly targeted therapies. Kirsten rat sarcoma viral oncogene (*KRAS*) and epidermal growth factor receptor (*EGFR*) are the most frequent and well-known mutated oncogenes in adenocarcinoma of the lung. Compared with other types of lung adenocarcinoma, lung adenocarcinoma with *EGFR* mutation shows a good response to treatment with *EGFR* tyrosine kinase inhibitors (TKIs), such as gefitinib and erlotinib [4, 5]. However, *KRAS* is still considered a nondrug target, and efforts to therapeutically target *KRAS* mutations have proved unsuccessful [6]. Indeed, *KRAS* has been proven to be a biomarker of resistance to *EGFR*-TKI treatment. In addition, previous studies have indicated that *KRAS* mutations are associated with worse survival, and these mutations are thought to be a negative prognostic marker in patients with lung cancer, especially patients with adenocarcinoma and early stage disease [7–11]. In the latest guideline (2018) from the College of American Pathologists/International Association for the Study of Lung Cancer/Association of Molecular Pathology, *EGFR* is indicated as a necessary testing gene for lung adenocarcinoma, and *KRAS* is a recommended testing gene, especially in cases where routine tests for *EGFR* show negative results [12].

Medical imaging – particularly computed tomography (CT) – is an essential noninvasive procedure for lung cancer diagnosis, staging and therapeutic response evaluation. The relationship between CT characteristics and lung cancer gene phenotypes has been a research area of particular interest, especially in relation to *EGFR* mutation [13, 14]. However, only a few studies have examined the correlation between the CT findings of lung adenocarcinoma and *KRAS* mutational status [15–18]. These studies showed that no or few inconsistent CT characteristics were associated with *KRAS* mutations. Furthermore, conventional CT imaging signs lack quantitative evaluation, making them vulnerable to subjective judgment. As a new, revolutionary CT imaging method, dual-energy spectral CT (DESCT) can improve material differentiation by using two different X-ray energy spectra [19, 20]. Compared to conventional mix-energy CT, DESCT scan can use a single tube with fast and dynamic kVp switching between 80 and 140 kVp X-rays during a single rotation and generates 101 monochromatic CT images in the range of 40 to 140 keV, as well as iodine/water-based density and effective atomic number images [21, 22]. Therefore, DESCT can provide multiple quantitative measurements, including the monochromatic CT number, the slope of the spectral Hounsfield unit (HU) curve (slope λ HU) based on monochromatic images, the iodine concentration (IC) based on iodine-based density images, the water concentration (WC) based on water-based density images, and the effective atomic number (effective Z) based on effective atomic number images. It has been proven that DESCT has potential applications in various clinical areas, including diagnostics in oncology [20, 23, 24]. Regarding lung cancer, DESCT has been employed in the differential diagnosis of cancers from benign lung nodules and the identification of lymph node metastases and has been used to distinguish histologic subtypes, such as adenocarcinoma and squamous cell carcinoma [25–31].

The occurrence of *KRAS* and *EGFR* mutations is mutually exclusive, and they exhibit many contrasting characteristics, such as clinical background and prognostic implications. To our knowledge, there has been scarce previous description of the DESCT characteristics of tumors with a *KRAS* mutation. We hypothesized that DESCT features can be used to distinguish *KRAS* mutations from *EGFR* mutations in lung adenocarcinoma. Therefore, we aimed to retrospectively explore potential differences in DESCT features between *KRAS* and *EGFR* mutations in a cohort of Chinese patients with lung adenocarcinomas.

## Materials and methods

### Patient selection

**Fig. 1:**
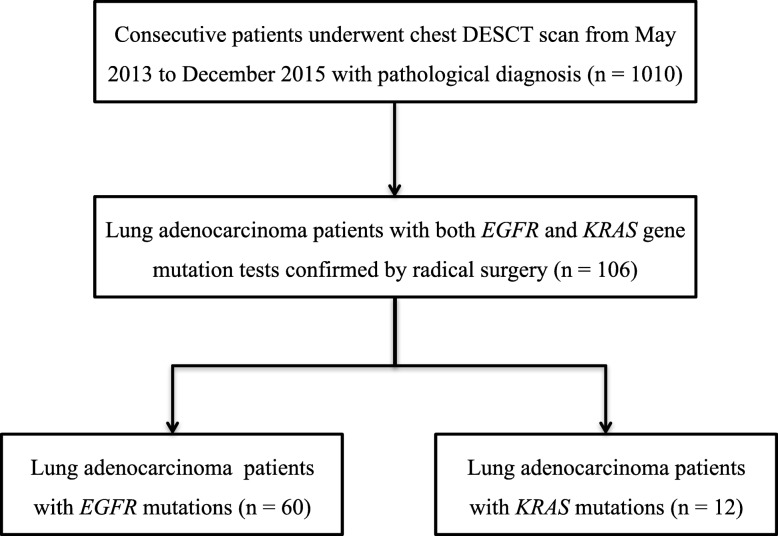
Flowchart depicting the patient selection

The study population was retrospectively selected from a prospectively collected and recorded database of information from patients who had lung nodules and masses and were undergoing pretreatment chest spectral DESCT from May 2013 to December 2015 at our institution. Inclusion criteria included being diagnosed with a cell type adenocarcinoma and having testing performed for *EGFR* and *KRAS* mutations after radical surgery at our institution (Fig. 1). The institutional ethics committee approved this study of prospectively collected data. Written informed consent for the use of clinical and imaging data for scientific and/or educational purposes was waived for this retrospective study.

### DESCT examination

All patients received a DESCT (Discovery CT 750 HD, GE Healthcare, USA) enhanced chest scan from the apex of the lung to the adrenal gland before treatment. The scan applied gemstone spectral imaging (GSI) mode protocol, whose tube voltage fast switching between 80 keV and 140 keV with a cycle of 0.5 ms. The other scanning parameters were as follows: tube current of 550 mA, tube rotation time of 0.6 s, collimator of 40 mm, helical mode with a pitch of 0.984, field of view (FOV) of large body, and slice thickness and interval for axial images of 1.25 mm and 0.8 mm. All patients were intravenously injected with contrast media (Ultravist 300; Bayer Pharma AG, Germany) using a power injector at a rate of 2.5 ml/s and volume of 85–100 ml (1.5 ml/kg of body weight). The scan acquisition was started after a delay of 35 s.

### DESCT image analysis

The CT of all lung nodules was evaluated visually by two experienced radiologists. The morphological nodule type included solid nodule (SN), part-solid nodule (PSN) or mixed ground-glass opacity (GGO), and nonsolid nodule (NSN) or pure GGO; NSN was defined as a hazy increased opacity of lung, with preservation of bronchial and vascular margins; PSN was defined as a combination of ground glass and solid attenuation, which obscures the underlying lung architecture on CT; NSN and PSN were both referred to as subsolid nodules (SSN) [32–34].

The original data acquired were reconstructed into monochromatic images. The reconstructed images were sent to a post processing workstation (Advantage Workstation 4.6, GE Healthcare, Milwaukee, WI), where GSI Viewer software was used to analyze the enhanced monochromatic data and determine quantitative parameters. For the axial image, a radiologist with 10 years of experience in CT diagnosis of chest tumors selected the axial CT slice that depicted the maximum diameter of the primary tumor and positioned the region of interest (ROI) at the center of the lesion manually. The ROI range was drawn with no less than 2/3 of the area of the lesion. Cavities, vacuoles, calcification, blood vessels and pulmonary atelectasis were avoided. Quantitative parameters measured included IC, WC, effective Z and slope λ HU, which was calculated as the difference between the CT number at 40 keV and that at 100 keV divided by the energy difference of 60 keV [slope λ HU = (CT number at 40 keV − CT number at 100 keV)/60]. The enhanced CT number at 70 keV was selected because the 120 kVp scanning in conventional polychromatic images has an average energy of approximately 70 keV in the GSI mode. To minimize the variations caused by the patient’s circulation status and the scanning times, the IC and WC of each lung lesion were normalized to the IC and WC of the descending aorta, respectively, at the T6 level to calculate a normalized IC (NIC; NIC = IC_lesion_/IC_aorta_) and a normalized WC (NWC; NWC = WC_lesion_/WC_aorta_). Finally, five types of quantitative data were obtained: CT number at 70 keV, slope λ HU, NIC, NWC and effective Z.

### Tumor pathologic characteristics and mutation analysis

All patient pathologies were confirmed by radical operative pathological examinations. All histologic and mutation analyses were performed on surgical specimens. Tumor histologic characteristics were classified on the basis of the 2015 World Health Organization criteria. The mutation status of *KRAS* and *EGFR* was examined by molecular pathological analysis.

### Statistical analysis

The patient clinical and DESCT characteristics of the study population are expressed as the means and standard deviations (X ± S) for continuous variables and as frequency or percentage for categorical variables. The normality of continuous variables was analyzed using one-sample Kolmogorov-Smirnov Z tests (K-S tests). Univariate analyses were performed to assess the difference in clinical and DESCT characteristics between patients with *KRAS* mutations and patients with *EGFR* mutations. A t test was used if the continuous data exhibited a normal distribution; the Mann-Whitney U test was used if the continuous data did not have a normal distribution. Categorical data were compared using chi-square (χ^2^) tests or Fisher’s exact tests. The significant factors in univariate analyses were identified as candidate covariates in logistic regression models with backward elimination of covariates, and the odds ratios (OR) were calculated. A receiver operating characteristic (ROC) curve was generated for *KRAS* mutation prediction according to each significant factor. Diagnostic capability was assessed by calculating the area under curve (AUC). *P* values < 0.05 were considered significant. The statistical analyses were performed using SPSS 19.0 (SPSS Inc., Chicago, IL) statistical software package.

## Results

A total of 72 patients with lung adenocarcinoma (30 males and 42 females; age 55.9 ± 11.6 years old) who underwent DESCT scanning and *EGFR* and *KRAS* testing were included in this study. According to the outcomes of gene testing, 60 patients had *EGFR* mutations (the *EGFR* group) and 12 patients exhibited *KRAS* mutations (the *KRAS* group).

### Clinical and nodule type of patients with *KRAS* mutations compared to those with *EGFR* mutations in lung adenocarcinomas

Patient clinical and DESCT characteristics are reported in Table 1. *KRAS* mutations were less common in nonsmoking people than *EGFR* mutations (33.3% vs 78.3%). Nodule type was significantly different between the two mutations (*P* = 0.035), and all *KRAS* mutation adenocarcinomas were SN tumors.

**Table 1:** Comparison between clinical and CT texture with *KRAS* and *EGFR* mutation status in lung adenocarcinoma

Characteristics	Total	*KRAS*	*EGFR*	*P* value
No. of patients	72	12	60	
Age (y)^a^	56.75 ± 10.13	58.75 ± 7.53	56.35 ± 10.58	0.458
Sex				0.054
Female	42 (58.3)	4 (33.3)	38 (63.3)	
Male	30 (41.7)	8 (66.7)	22 (36.7)	
Smoking				**0.002**
Never smoked	55 (76.4)	4 (33.3)	47 (78.3)	
Smoker	17 (23.6)	8 (66.7)	13 (21.7)	
Location				0.521
Central	2 (2.8)	0 (0.0)	2 (3.3)	
Peripheral	70 (97.2)	12 (100.0)	58 (96.7)	
T stage				0.066
T_1–2_	68 (94.4)	10 (83.3)	58 (96.7)	
T_3–4_	4 (5.6)	2 (16.7)	2 (3.3)	
N stage				0.702
N_0_	47 (65.3)	9 (7.5)	38 (63.3)	
N_1–2_	25 (34.7)	3 (2.5)	22 (36.7)	
Maximum diameter^b^	2.89 ± 1.29	3.17 ± 1.38	2.83 ± 1.29	0.666
CT texture feature				**0.035**
SN	56 (77.8)	12 (100.0)	44 (73.3)	
SSN (PSN and NSN)	16 (22.2)	0 (0.0)	16 (26.7)	

Note. Values are mean ± standard deviation or number (percentage)

^a^Quantitative data exhibited normal distribution and T test was applied

^b^Quantitative data did not exhibit normal distribution and Mann-Whitney U test was applied

*P* < 0.05 indicates significant difference. Significant *P* values are in bold

*SN* solid nodule, *SSN* Subsolid nodule, *NIC* Normalized iodine concentration, *NWC* Normalized water concentration, *Slope λ HU* the slope of the spectral Hounsfield unit curve, *Effective Z* effective atomic number

### Influence of nodule type on the quantitative parameters from DESCT

The mean values of the CT number at 70 keV, slope λ HU, NIC, NWC, and effective Z were significantly different in SN tumors compared to SSN tumors, as shown in Table 2. There was no statistically significant difference in the effective Z between SN and SSN tumors, although the mean value in SSN was lower than that in SN (6.69 vs. 8.45).

**Table 2:** Association of CT texture type and DESCT features

Characteristics	Total	SN	SSN	*P* value
No. of patients	72	56	16	
DESCT quantitative parameter				
CT number at 70 keV^a^	−5.25 ± 139.64	47.39 ± 23.04	− 189.49 ± 209.28	**0.000**
Slope λ HU^a^	1.95 ± 1.03	1.71 ± 0.92	2.78 ± 0.98	**0.000**
NIC^a^	0.22 ± 0.12	0.19 ± 0.12	0.28 ± 0.11	**0.004**
NWC^a^	0.93 ± 0.14	0.99 ± 0.02	0.74 ± 0.19	**0.000**
Effective Z^a^	8.06 ± 1.82	8.45 ± 0.43	6.69 ± 3.53	0.866

Note. Values are mean ± standard deviation or number

^a^Quantitative data did not exhibit normal distribution and Mann-Whitney U test was applied

*P* < 0.05 indicates significant difference; Significant *P* values are in bold

*SN* solid nodule, *SSN* Subsolid nodule, *NIC* Normalized iodine concentration, *NWC* Normalized water concentration, *Slope λ HU* the slope of the spectral Hounsfield unit curve, *Effective Z* effective atomic number

### Clinical and quantitative DESCT parameters of patients with *KRAS* mutations compared to those with *EGFR* mutations in solid lung adenocarcinoma

**Fig. 2:**
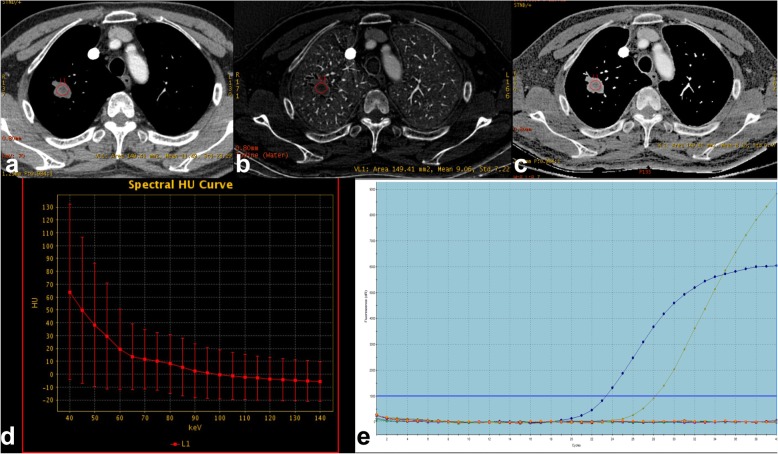
Male, 73 years old, lung adenocarcinoma with *KRAS* mutation. (**a**) A DESCT 70 keV image showed a solid nodule in the middle lobe of the right lung; the CT number at 70 keV was 11.49 Hu. (**b**) An iodine-based material-decomposition image reveals that the iodine concentration (IC) of the nodule is 9.06 μg/cm3 (L1). The IC of the aorta is 77.24 μg/cm3. The normalized IC (NIC) of this lung adenocarcinoma is 0.12 (9.06/77.24). (**c**) The effective Z material-decomposition image shows that the effective Z of the nodule is 8.15. (**d**) The graph shows the spectral HU curve of the nodule. Slope λ HU is 1.01. (**e**) The molecular pathological results showed *KRAS* mutations

**Fig. 3:**
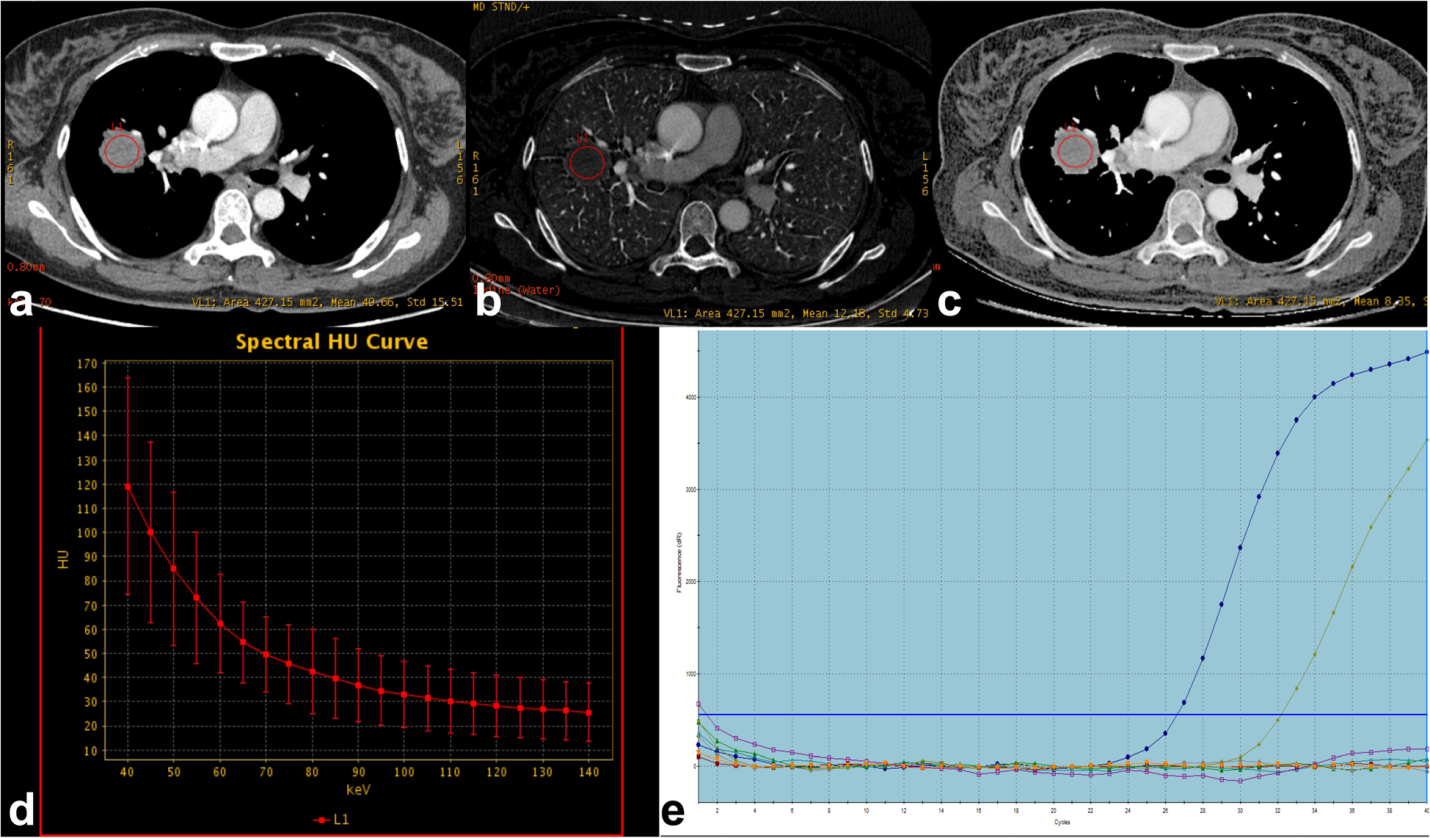
Female, 39 years old, lung adenocarcinoma with *EGFR* mutation. (**a**) A DESCT 70 keV image showed a solid nodule in the inferior lobe of the left lung. The CT number at 70 keV was 49.66 HU. (**b**) An iodine-based material-decomposition image shows that the iodine concentration (IC) of the nodule is 12.18 μg/cm3; the IC of the aorta is 76.23 μg/cm3; the normalized IC (NIC) of this lung adenocarcinoma is 0.16 (12.18/76.23). (**c**) The effective Z material-decomposition image shows that the effective Z of the nodule is 8.35. (**d**) The graph shows the spectral HU curve of the nodule; slope λ HU is 0.82. (**e**) The molecular pathological results showed *EGFR* mutations

Because nodule type has obviously impact on DESCT quantitative parameters and all *KRAS* mutation adenocarcinomas were SN tumors, to make the measurement comparable, we deleted imaging data of the *EGFR* mutation group with SSN tumors before comparing differences between the two groups (*n* = 12 to *n* = 44). The clinical and DESCT characteristics of solid lung adenocarcinoma are reported in Table 3. For DESCT quantitative parameters, the CT number at 70 keV, slope λ HU, NIC, and effective Z values differed significantly between the *KRAS* and *EGFR* groups (*P =* 0.006, 0.017*,* 0.013 and 0.010, respectively) (Figs. 2, 3).

**Table 3:** Comparison between clinical and DESCT characteristics with *KRAS* and *EGFR* mutation status in solid lung adenocarcinoma

Characteristics	Total	*KRAS*	*EGFR*	*P* value
No. of patients	56	12	44	
Age (y)^a^	55.98 ± 10.37	58.75 ± 7.53	55.23 ± 10.97	0.301
Sex				0.149
Female	29 (51.8)	4 (33.3)	25 (56.8)	
Male	27 (48.2)	8 (66.7)	19 (43.2)	
Smoking				**0.012**
Never smoked	36 (64.3)	4 (33.3)	32 (72.7)	
Smoker	20 (35.7)	8 (66.7)	12 (27.3)	
Location				0.452
Central	2 (3.6)	0 (0.0)	2 (4.5)	
Peripheral	54 (96.4)	12 (100.0)	42 (95.5)	
T stage				0.148
T_1–2_	52 (92.9)	10 (83.3)	42 (95.5)	
T_3–4_	4 (7.1)	2 (16.7)	2 (4.5)	
N stage				0.158
N_0_	32 (57.1)	9 (7.5)	23 (52.3)	
N_1–2_	24 (42.9)	3 (2.5)	21 (47.7)	
Maximum diameter^b^	3.15 ± 1.32	3.17 ± 1.38	3.14 ± 1.32	0.742
DESCT quantitative parameter				
CT number at 70 keV^a^	47.39 ± 23.04	31.52 ± 22.26	51.71 ± 21.51	**0.006**
Slope λ HU^b^	1.71 ± 0.92	1.17 ± 0.77	1.85 ± 0.91	**0.017**
NIC^b^	0.198 ± 0.12	0.14 ± 0.09	0.21 ± 0.12	**0.013**
NWC^b^	0.989 ± 0.02	0.98 ± 0.02	0.99 ± 0.02	0.239
Effective Z^a^	8.45 ± 0.43	8.17 ± 0.39	8.52 ± 0.41	**0.010**

Note. Values are mean ± standard deviation or number (percentage)

^a^Quantitative data exhibited normal distribution and T test was applied

^b^Quantitative data did not exhibit normal distribution and Mann-Whitney U test was applied

*P* < 0.05 indicates significant difference; Significant *P* values are in bold

*NIC* Normalized iodine concentration, *NWC* Normalized water concentration, *Slope λ HU* the slope of the spectral Hounsfield unit curve, *Effective Z* effective atomic number

**Fig. 4:**
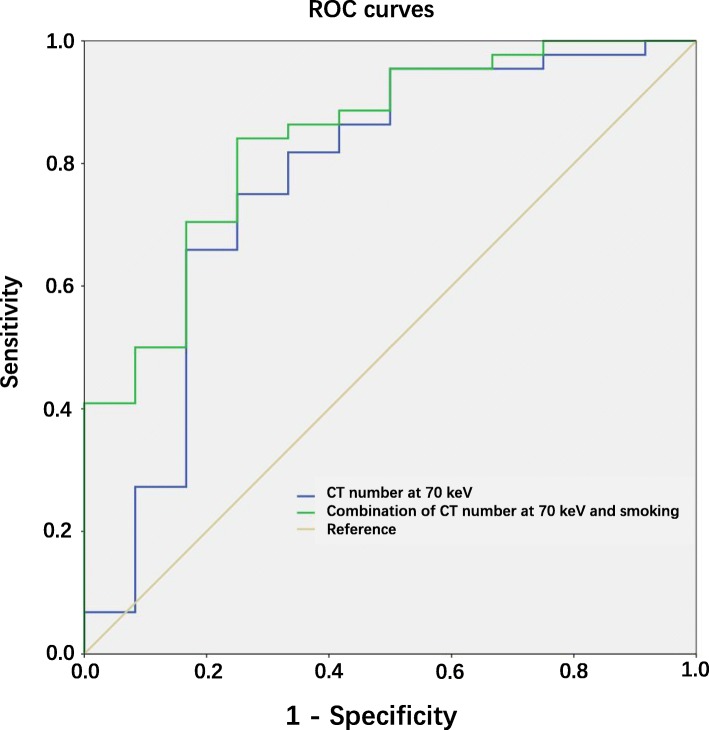
Graph shows the receiver operating characteristic (ROC) curve for discrimination of patients with *KRAS* mutations from those with *EGFR* mutations at DESCT (CT number at 70 keV) including and not including smoking in solid lung adenocarcinoma. The area under curve (AUC) of CT number at 70 keV is 0.771 with the cutoff point of 38.47 HU. The AUC of combination of CT number at 70 keV and smoking is 0.841 with the cutoff point of 2.72

Multivariate analyses evaluating smoking, sex, CT number at 70 keV, NIC, effective Z, and slope λ HU showed that smoking (OR = 7.421, *P* = 0.016) and CT number at 70 keV (OR = 0.938, *P* = 0.009) were two independent prognostic factors for *KRAS* mutations compared to *EGFR* mutations in solid lung adenocarcinoma (Table 4). The AUC of CT number at 70 keV is 0.771 (95% CI: 0.597–0.945, *P* = 0.004) with the cutoff point of 38.47 HU. Based on this multivariate analysis, the two significant factors (CT number at 70 keV and smoking history) were combined to determine the predictive value to differentiate *KRAS* and *EGFR* mutations. The AUC of combining the two factors was 0.841 (95% CI: 0.717–0.965, *P* < 0.001) with the cutoff point of 2.72 (Fig. 4).

**Table 4:** Multivariable Analysis of DESCT and Clinical Features Predicting the Presence of *KRAS* Mutation Compared to *EGFR* Mutation in Solid Lung Adenocarcinoma

Characteristics	OR	95% CI	*P* value
Smoking			0.016
Never smoked	Reference	NA	
Smoker	7.421	1.451–37.948	
CT number at 70 keV	0.938	0.894–0.984	0.009

Note. *NA* not applicable. *OR* odd ratio. *CI* confidence interval

## Discussion

Few studies have investigated conventional CT features and *KRAS* mutations in lung adenocarcinoma. Previous studies by Glynn et al. [15] did not find any conventional CT characteristics associated with *KRAS* mutations in patients with lung adenocarcinoma. Although some other studies showed that size, spiculation sign, and air bronchogram sign may be related to *KRAS* mutations, the results were quite inconsistent [16–18]. These negative or inconsistent results may reflect the limitations of conventional CT imaging signs, which lack a quantitative index and are unstable due to subjective judgment. In contrast, it is now widely recognized that the GGO ratio is significantly higher in tumors with *EGFR* mutations [13, 35, 36]. This phenomenon may be because *EGFR* mutations appear more frequently in lepidic predominant adenocarcinomas, which are associated with better outcomes [35, 37]. In this study, the SSN rate in tumors with *EGFR* mutations was higher than that in *KRAS* mutations (26.7% vs. 0%), and all *KRAS* mutation tumors were solid.

Given the difference in SSN that was observed between the *KRAS* and *EGFR* groups, we also studied the relationship between nodule type and DESCT quantitative parameters, which also has scarce been reported previously. Our results showed that all DESCT quantitative parameters, except effective Z, differed between SSN tumors and SN tumors. Effective Z was lower in SSN than SN, and although no statistically significant difference was observed, more sample size research is needed. The SSN contains an extremely low air attenuation, which result in low CT number at 70 keV. It is worthwhile to note that NIC and Slope λ HU of SSN were higher than SN on the contrary. This results suggest that NIC and Slope λ HU can hardly be affected by the low air attenuation in SSN, and the reason maybe the relatively small size and rich blood vessels or volume in the early stage tumor [38].

To eliminate the impact of SSN on DESCT quantitative parameters, and since the *KRAS* mutation adenocarcinoma are all SN as well, we deleted imaging data of SSN and then compared the difference between the two groups (*KRAS n* = 12 to *EGFR n* = 44). The results showed that the CT number at 70 keV, slope λ HU, NIC, and effective Z were significantly different between solid lung adenocarcinomas with *KRAS* and *EGFR* mutations. *KRAS* mutations in lung adenocarcinoma have special pathological features. In terms of the histological type, *KRAS* mutations are associated more with mucinous adenocarcinoma or lung cancer with goblet cell morphology than with nonmucinous adenocarcinoma [39–42]. On the other hand, studies have shown that in addition to cancer genesis and development, *EGFR* also plays important roles in stimulating angiogenesis through very complicated biological processes [43, 44]. We speculate that the DESCT findings might correlate with the underlying pathologic appearance. The mucus produced in *KRAS* mutation lung adenocarcinoma and the rich blood supply of *EGFR* mutation lung adenocarcinoma may result in the lower quantitative value with *KRAS* mutations compared to *EGFR* mutations.

A relationship between *KRAS* mutational status and lung CT image features could improve the accuracy of medical decisions. Multivariate logistic analysis combining clinical and DESCT characteristics showed that CT value at 70 keV and smoking were the two independent factors potentially able to predict the presence of *KRAS* mutations from *EGFR* mutations in solid lung adenocarcinomas. The combination of CT number at 70 keV with smoking history was a powerful tool to differentiate *KRAS* and *EGFR* mutations, which could be used to aid in clinical diagnosis in the future. The ROC obtained by combining these significant factors also showed a relatively high predictive value for identifying *KRAS* mutations (AUC = 0.841, 95% CI: 0.717–0.965). This finding suggests that combining clinical and DESCT characteristics can be recommended for use to differentiate *KRAS* and *EGFR* status in solid lung adenocarcinomas.

The prevalence of *KRAS* mutations is much lower in East Asian patients than in Western patients (8.3% vs. 32%, respectively) [45, 46]. Our study showed a *KRAS* mutation prevalence of 11.3% (12/106) in this population. In a previous study, *KRAS* mutations were more frequent in smokers and male patients than *EGFR* mutations [47]. In the same study, smoking history was found to be a significant determinant, while gender was a confounding factor [47]. In this study’s analysis of clinical characteristics, smoking history was significant factor in both univariate and multivariate analyses, which is consistent with previous work. The *KRAS* mutation was also more frequent in males than the *EGFR* mutation, but this gender difference was not significant (*P* = 0.054).

Although histological and immunohistochemical analyses have been accepted as the reference standard, identification of the relationship between DESCT quantitative measurements and *KRAS* status could help determine the molecular categories of lung adenocarcinoma. First, histological and immunohistochemical analyses of biopsies or surgical specimens is an invasive method, and it has also been well documented that diagnostic errors are common [48, 49]. Hence, additional diagnostic information can help improve accuracy. Second, compared with molecular technologies, routine imaging can provide a more comprehensive view of the entire tumor and can be used on an ongoing basis to monitor relapse after surgery much less invasively. This benefit is even more critical in larger tumors, which can exhibit intratumor genomic heterogeneity [50]. Third, the relationship may suggest a greater need for blinded targeted therapies for the patients who cannot undergo histological sampling.

This study is the first to describe the imaging differences between lung cancer patients with *KRAS* and *EGFR* mutations using DESCT according to our knowledge. The present study also has several limitations. First, the retrospective single-center design has various potential biases. Second, the enrolled sample size was relatively small, especially for patients with *KRAS* mutations. Therefore, studies should be conducted with larger sample sizes to examine the precise characteristics of these mutations in the future.

## Conclusions

In conclusion, the SN proportion was higher with *KRAS* than *EGFR* mutations and all *KRAS* mutation adenocarcinomas were SN tumors. DESCT features, especially CT number at 70 keV, can be an image biomarker to help distinguish *KRAS* and *EGFR* mutations in solid lung adenocarcinoma. Combining DESCT-based features with clinical variables – such as CT value at 70 keV with smoking history – is a promising approach for improving the discrimination of *KRAS* mutations from *EGFR* mutations in solid lung adenocarcinoma.

## Data Availability

The datasets used and/or analyzed during the current study are available from the corresponding author on reasonable request.

## Abbreviations

DESCT: Dual-energy spectral computed tomography
Effective Z: Effective atomic number
*EGFR*: Epidermal growth factor receptor
FISH: Fluorescence in situ hybridization
GGO: Ground-glass opacity
GSI: Gemstone spectral imaging
IHC: Immunohistochemistry
*KRAS*: Kirsten rat sarcoma viral oncogene homolog
NIC: Normalized iodine concentration
NSN: Nonsolid nodule
NWC: Normalized water concentration
Slope λ HU: Slope of the spectral Hounsfield unit curve
SN: Solid nodule
SSN: Subsolid nodule
